# An Acute Application of Cerebellar Transcranial Direct Current Stimulation Does Not Improve Motor Performance in Parkinson’s Disease

**DOI:** 10.3390/brainsci10100735

**Published:** 2020-10-14

**Authors:** Lidio Lima de Albuquerque, Milan Pantovic, Mitchel Clingo, Katherine Fischer, Sharon Jalene, Merrill Landers, Zoltan Mari, Brach Poston

**Affiliations:** 1 Department of Kinesiology and Nutrition Sciences, University of Nevada Las Vegas, Las Vegas, NV 89154, USA; milan.pantovic@unlv.edu (M.P.); fische74@unlv.nevada.edu (K.F.); sharon.jalene@unlv.edu (S.J.); brach.poston@unlv.edu (B.P.); 2 School of Medicine, University of Nevada Las Vegas, Las Vegas, NV 89102, USA; clingom@unlv.nevada.edu; 3 Department of Physical Therapy, University of Nevada Las Vegas, Las Vegas, NV 89154, USA; merrill.landers@unlv.edu; 4 Movement Disorders Program, Cleveland Clinic Lou Ruvo Center for Brain Health, Las Vegas, NV 89106, USA; mariz@ccf.org

**Keywords:** Parkinson’s disease, transcranial direct current stimulation, motor skill, cerebellum, cerebellar stimulation

## Abstract

Transcranial direct current stimulation of the cerebellum (c-tDCS) improves motor performance in young and old adults. Based on the cerebellar involvement in Parkinson’s disease (PD), c-tDCS could have potential to improve motor function in PD. The purpose was to determine the effects of c-tDCS on motor performance in PD while participants were on medications. The study was a randomized, double-blind, SHAM-controlled, between-subjects design. Twenty-two participants with PD were allocated to either a c-tDCS group or a SHAM group. All participants completed one experimental session and performed two motor tasks with their most affected hand in a Baseline condition (no stimulation) and an Experimental condition. The motor tasks were a visuomotor isometric precision grip task (PGT) and a rapid arm movement task (AMT). The primary dependent variables were force error and endpoint error in the PGT and AMT, respectively. There were no significant differences in force error or endpoint error in the Experimental condition between the c-tDCS and SHAM groups. These results indicate that an acute application of c-tDCS does not enhance motor performance in hand and arm tasks in PD. Longer-term c-tDCS application over multiple days may be needed to enhance motor function in PD.

## 1. Introduction

Parkinson’s disease (PD) is a progressive, neurodegenerative disorder that is characterized by debilitating motor symptoms including tremor, rigidity, postural instability, and bradykinesia. The current pharmaceutical, surgical, and other management strategies for PD are directed towards relieving the symptoms associated with the disease. Levodopa, often combined with other medications represents the standard treatment for PD, but their efficacy diminishes over time [1] and leads to side effects such as dyskinesia. For advanced PD, advanced treatments such as deep brain stimulation are often considered and can substantially improve motor performance and quality of life [2]. However, deep brain stimulation is associated with surgical contraindications, high costs, neuropsychiatric side effects, and is not effective in treating non-motor PD symptoms [3]. Physical exercise is also commonly prescribed in PD primarily based on animal studies [4,5], and literature reviews have concluded that exercise is mild to moderately effective in improving many aspects of motor function in PD [6,7]. While several forms of exercise [8,9] can induce clinically significant motor improvements in PD, the most successful strategy to improve motor function would likely entail pairing adjunctive therapies with rehabilitation to enhance or complement the effects of existing treatments. Transcranial direct current stimulation (tDCS) is a form of non-invasive brain stimulation that when applied to the primary motor cortex (M1-tDCS) has shown the ability to increase cortical excitability and improve motor performance in healthy adults [10] and in PD [11]. These positive effects on motor performance are thought to be mediated by the changes in excitability in acute conditions and by modulation of GABAergic and glutamatergic synaptic connections in longer-term conditions [12]. In addition, tDCS has realistic potential for implementation into clinical practice [13], remote supervision paradigms [14], and into home-based care [15]. Furthermore, tDCS has several practical advantages that make it compatible with other therapies such as portability, safety, ease of administration, ability to be delivered during motor activities, and low cost [13]. Although the vast majority of studies in healthy adults and PD have utilized M1-tDCS, increasing evidence suggests that tDCS delivered to the cerebellum (c-tDCS) can also significantly improve motor performance [16,17,18]. However, all of these studies have been conducted in young and old adults, even though the cerebellum is now recognized to play a significant role in the pathology of PD [19,20]. With the current PD therapies affected by numerous limitations, there is heightened need to develop additional treatment options, including novel treatment modalities.

PD has historically been viewed primarily as a basal ganglia disorder due to the well-known loss of dopaminergic cells in the substantia nigra pars compacta, which leads to reductions in striatal dopamine and motor impairments. However, several studies have found altered functional activity in many other important brain areas, especially in M1 and the cerebellum. Accordingly, it is assumed that cerebellar pathology contributes to impaired motor function in PD. This provides a theoretical basis for the investigation of c-tDCS as a potential intervention in the disease [19,20]. Furthermore, several other specific research findings support this rationale: (1) c-tDCS improves motor performance in young [16,18] and older healthy adults [17] and M1-tDCS improves performance in these populations [10,21] and in PD [11]; (2) increased cerebellar activity compensates for basal ganglia dysfunction in PD and cerebellar stimulation may enhance this process [20]; (3) tDCS exerts remote effects on connected brain regions not stimulated directly and formerly unidentified reciprocal pathways have been found between the cerebellum and basal ganglia [22]; and (4) tDCS efficacy scales with age [21] and the level of impairment in motor disorders [23], which is relevant because the majority of individuals with PD are over the age of 60. Despite these observations, no studies have examined the effects of c-tDCS on fine motor performance of the hand and arm in PD, although a study by Workman and colleagues found that c-tDCS could improve balance in PD [24]. 

The purpose of the study was to determine the effects of c-tDCS on motor performance in PD while on medications. This was accomplished by having participants perform two motor tasks with their most affected hand in a Baseline condition and in an Experimental condition. Most importantly, one group of participants received c-tDCS during performance of the motor tasks in the Experimental condition, whereas the other group received SHAM stimulation. Thus, this allowed for the examination of whether c-tDCS applied simultaneous with motor task execution can enhance task performance. The two motor tasks selected for the study included a visuomotor isometric precision grip task (PGT) and a rapid arm movement task (AMT). These tasks were chosen because it has been shown that there is strong cerebellar involvement in the motor control and execution of both tasks [25,26,27,28]. Based on previous c-tDCS studies in young [16,18] and especially old adults [17], it was hypothesized that c-tDCS would enhance performance in the two motor tasks compared to SHAM stimulation. 

## 2. Materials and Methods

### 2.1. Participants

Twenty-two individuals with PD (12 males, 10 females; mean age: 71.3 ± 8.6 years) volunteered to participate in the study. All participants were diagnosed by a movement disorders neurologist according to standard clinical diagnostic criteria [29]. Participants were free of any other neurological disorder, did not have an uncontrolled medical condition, and did not meet exclusion criteria for non-invasive brain stimulation studies. Twenty of the participants were primarily right-side affected and right-hand dominant, while two participants were primarily left-side affected and left-hand dominant. The participants provided written informed consent, the protocol (Number 743597) was approved by the IRB at the University of Nevada Las Vegas, and the research was conducted according to the Declaration of Helsinki.

### 2.2. Experimental Design and Procedures

**Figure 1:**
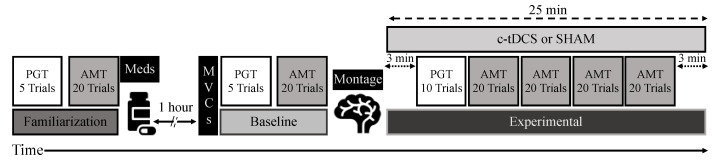
Schematic representation of the experimental protocol that included a familiarization (5 precision grip task (PGT) trials; 20 arm movement task (AMT) trials), medication ingestion followed by 1 h of rest, 3 maximal voluntary contraction (MVC) trials, a Baseline condition (5 PGT trials; 20 AMT trials), cerebellar transcranial direct current stimulation (c-tDCS) montage placement, and an Experimental condition (25 min of either c-tDCS or SHAM stimulation concurrent with 10 PGT trials and 4 blocks of 20 AMT trials).

The study utilized a randomized, double-blind, SHAM-controlled, between-subjects design. Participants were allocated to either a c-tDCS group (*n* = 11) or a SHAM stimulation group (*n* = 11) and completed a single 2–2.5 h experimental session. All participants initially reported to the lab in the morning after a 12-h overnight medication withdrawal (practically defined-OFF condition) but performed the experimental interventions and testing on medications (see below). At the beginning of the session, participants were familiarized with all experimental procedures. This included visual demonstrations by one of the investigators of all the motor tasks associated with the study. During this time, participants were required to demonstrate that they understood the task requirements and the visual feedback provided by the computer programs during the tasks. Subsequently, they completed 5 familiarization trials of the PGT and 20 familiarization trials of the AMT. After the familiarization, the following procedures were performed in the order described: (1) participants ingested their PD medications and sat quietly for one hour to allow the medications to take effect; (2) three maximal voluntary contractions (MVCs) were completed; (3) the baseline condition was performed and involved 5 trials of the PGT and 20 trials of the AMT; (4) the c-tDCS electrode montage was placed on the head; (5) the stimulator was turned on for 3 min while participants sat quietly; (6) the experimental condition was completed and involved 10 trials of the PGT and 4 blocks of 20 trials of the AMT performed simultaneously with the application of c-tDCS; and (7) the stimulator kept running for any time remaining in the overall 25 min stimulation period (Figure 1).

### 2.3. Experimental Tasks

#### 2.3.1. MVCs

A total of three MVC trials were executed using the same experimental arrangement and hand posture as in the PGT task. Participants were required to produce maximum force in the shortest possible time and to hold the maximum for 3–5 s as described in previous studies [30,31]. The MVC task was used to determine the reference value to compute the target forces for the PGT.

#### 2.3.2. PGT 

The experimental setup for the PGT was similar to the arrangement used in previous studies [31,32]. Participants sat in a chair and placed their dominant-primarily affected arm on a table located on one side of the chair. The forearm was positioned on the table with the wrist in a neutral position, the shoulder abducted (45°), the elbow flexed (90°), and with the hand in a semi-supinated posture. The PGT required participants to match a sine wave target template that scrolled across a computer monitor. This was accomplished by producing isometric force using the index finger and thumb in a precision grip against a custom-made grip manipulandum equipped with force transducers. Participants were instructed to match their force trace to the target template as accurately as possible for the duration of each PGT trial. The minimum and maximum values of the sine wave were 5% and 25% of the MVC force, whereas the sine wave frequency was set to 1 Hz. A single PGT trial involved matching the template for a duration of 30 s followed by 30 s of rest. In the familiarization and in the Baseline condition, the PGT was performed without concurrent stimulation. In contrast, the PGT was performed simultaneously with c-tDCS or SHAM stimulation during the Experimental condition. Finally, note that the stimulator was turned on for 3 min [18] as participants sat quietly prior to the start of the first PGT trial (Figure 1). 

The PGT was selected as a motor task for three interrelated reasons. First, precision grip tasks have been successfully employed in several M1-tDCS studies [33,34,35] and a c-tDCS study in young adults [36]. Furthermore, a study (manuscript in preparation) involving M1-tDCS in PD that was recently completed in our lab utilized the PGT. All of the aforementioned studies found that tDCS evokes substantial increase in force accuracy in these precision grip tasks. Thus, there is a high probability that increases in performance due to c-tDCS can be evoked in the PGT. Second, the precision grip is a complex, multi-joint functional task that is employed frequently in tasks of daily living; (3) numerous studies have shown that there is a high level of cerebellar involvement in visuomotor tracking tasks [28]. In addition, the PGT is relevant to PD for at least two important reasons. First, impairments in force generation and relaxation are well-characterized in PD in isometric precision grip tasks and highly dependent on basal ganglia function [37,38,39]. Second, the PGT involves strict force accuracy of force production requirements and force accuracy is usually lower in PD compared to age-matched controls [40].

#### 2.3.3. AMT

The AMT was performed in a nearly identical manner and experimental arrangement as in a previous study in young and old adults [41]. Briefly, participants executed the AMT with the dominant, primarily affected arm on a digitizer tablet with a digitizer pen while receiving visual feedback of the task on a computer monitor linked to the tablet. Participants were instructed to perform the AMT as fast and as accurately as possible using a single movement that required elbow extension and shoulder flexion. The movements were performed from a home circle to a very small target circle of 0.5 cm in diameter located at a relatively long distance (20 cm) away from the participant. Once the pen was stationary and the participants had assumed the correct starting posture, a “GO” signal was given by a customized script that controlled the experiment. Participants then performed the movement at their convenience (no reaction time component). Furthermore, the script did not provide participants with visual feedback of the cursor movement (trajectory) during the trial, but they receive visual feedback of their endpoint performance relative to the target, which was provided 1 s after each trial for a duration of 3 s. Participants were directed to endeavor to minimize endpoint error distance on each successive trial. The AMT was performed without concurrent stimulation in the familiarization and in the Baseline condition. In contrast, the AMT was performed simultaneously with c-tDCS or SHAM stimulation during the Experimental condition. Finally, note that the stimulator was kept on after completion of the last AMT trial block (usually a time frame of 1–3 min) until the 25 min stimulation period elapsed (Figure 1). 

The AMT was selected as a motor task due to the strong cerebellar involvement in several related aspects of movement control related to this task [25,26,27,28]. First, it is an unconstrained, multi-joint movement entailing the prediction, regulation, and exploitation of joint interaction torques. Second, it involves the precise timing of agonist and antagonist muscle bursts to attain endpoint accuracy. Third, trial-to-trial performance in the AMT is highly dependent on the ability to detect and correct for endpoint errors on successive trials. In addition, the AMT is relevant to PD for several reasons. First, multi-joint arm movement control impairments are well-characterized in PD [42]. Second, movement velocity is one aspect of the AMT and this would reflect on the ability of c-tDCS to address bradykinesia in PD. Third, the AMT involves strict endpoint accuracy requirements and movement accuracy is usually lower in PD compared to age-matched controls. 

#### 2.3.4. c-tDCS

A NeuroConn DC Stimulator Plus/MR delivered tDCS through two rubber electrodes (5 cm × 5 cm) that were encased in saline soaked sponges and secured in place by two rubber straps. Anodal c-tDCS was applied over the cerebellum ipsilateral to the primarily affected hand with the anode 3 cm lateral to the inion and the cathode over the ipsilateral buccinator muscle [43]. The stimulation duration was 25 min and the current strength was set to 2 mA. These c-tDCS parameters produced substantial and immediate increases in motor performance in young and old adults in prior studies [16,17,18]. Note that the stimulator was turned on for 3 min while participants sat quietly before performing the first PGT trial [18]. In the SHAM stimulation condition, the current was ramped up and down over 30 s, which is the most widely utilized SHAM stimulation protocol in the literature [44]. The tDCS device settings were programmed by an investigator who did not partake in either the data collection sessions or the data analysis portion of the project. Similarly, the researchers who conducted the experiments and analyzed the data were blind to the experimental condition in each experiment. 

### 2.4. Data Analysis

The dependent variables were the force error in the PGT and the endpoint error and movement velocity in the AMT. All PGT data were collected in Spike2 software (Cambridge Electronic Design, Cambridge, UK) and analyzed in custom-written Spike2 scripts. AMT data were collected with Movalyzer software (Neuroscript, Tempe, AZ, USA) and analyzed using custom-written Matlab code [41]. Force error was computed as the average error in force over each 30 s trial of the PGT. Specifically, the difference (absolute value) between the force produced and the target force associated with the template was calculated at each sampling point for the entire 30 s trial and then the average of these values was taken. Thus, this average represented the average force error for one 30 s trial. The grand averages of the trials were then taken for the Baseline and Experimental conditions. Accordingly, the average of the 5 trials in the Baseline condition and the average of the 10 trials in the Experimental condition were taken as the final force error values for analysis. For the AMT, the endpoint error was calculated using the Pythagorean Theorem as in previous studies [41,45]. Thus, the *x*, *y* coordinates of the final endpoint of the pen and the center of target were used to determine the shortest distance between these two points. Movement velocity was calculated and quantified as the average velocity according to previous methods [41]. The average of the 20 trials in the Baseline condition and the average of the 80 trials (4 blocks of 20 trials) in the Experimental condition were taken as the final endpoint error and average velocity values for analysis.

### 2.5. Statistical Analysis

The UPDRS scores in the OFF medication state and the levodopa equivalent daily dose (LEDD) between groups were compared with unpaired t-tests, whereas Hoehn and Yahr scores were evaluated with a Chi Square Test. Two-way ANOVAs (2 group (c-tDCS, SHAM) × 2 condition (Baseline, Experimental)) with repeated measures on condition were used to compare force error in the PGT as well as endpoint error and movement velocity in the AMT, respectively. Post-hoc tests (Fisher LSD) were used to locate differences among pairs of means when appropriate. The significance level was set at α < 0.05 and data are indicated as means ± standard errors in the figures.

## 3. Results

**Figure 2:**
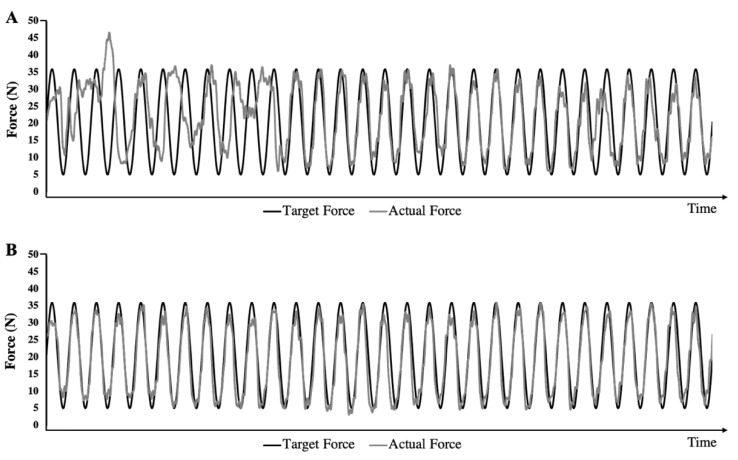
Representative precision grip task (PGT) trials illustrate the significant decline in force error that occurred between the two conditions. For this representative participant, the reduction in force error was 4.22 N between these two trials. (**A**) The force time profile for a single trial performed in the Baseline condition. (**B**) The force time profile for a single trial performed in the Experimental condition.

The UPDRS scores were 24.7 ± 5.7 for the c-tDCS group and 28.4 ± 12.1 for the SHAM group and the difference between groups was not statistically significant (*p* = 0.377). LEDD was also similar between the two groups (c-tDCS group = 584.8 ± 516.2, SHAM group 468.5 ± 193.7; *p* = 0.63). The Hohn and Yahr scores were 2.3 ± 0.65 for the c-tDCS group and 2.0 ± 0.63 for the SHAM group and the Chi Square Test was not significant (*p* = 0.310). Representative PGT data for the Baseline and Experimental conditions for one participant are shown in Figure 2. For the two-way ANOVAs, all of the assumptions were met, and no corrections were made. In regard to the assessment of normality of the data, the Shapiro–Wilk test was used due to the relatively small sample size. None of the Shapiro–Wilk tests were significant for the force error or movement velocity. Furthermore, the Shapiro–Wilk test was only significant (0.046) for the distribution of data for the SHAM group in the Experimental condition and not for the other three sets of data (factor levels). Therefore, we decided to perform parametric statistics for not only force error and movement velocity, but also for endpoint error. 

### 3.1. PGT

**Figure 3:**
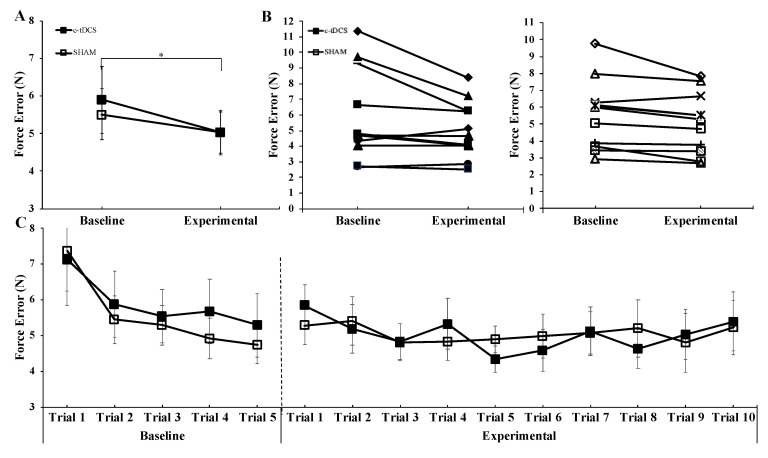
Force error in the precision grip task (PGT) for the cerebellar transcranial direct current stimulation (c-tDCS) (closed squares) and SHAM groups (open squares) in the Baseline and Experimental conditions. (**A**) The force error was not significantly different between the c-tDCS and SHAM groups (*p* = 0.861), but it was significantly lower in the Experimental condition compared to the Baseline condition (*p* = 0.005). * indicates a significant difference between the Baseline and Experimental conditions. (**B**) Individual PGT participant data corresponding to the data in Panel (A) for the 11 participants in the c-tDCS group and 11 participants in the SHAM group. (**C**) For illustrative purposes, the individual PGT trials corresponding to the average data presented in Panel (A) are depicted. It can be seen that there is no trend for PGT performance to improve with trial number to a greater extent in the c-tDCS group compared with the SHAM group due to the accumulating stimulation time.

The group × condition interaction (F_(1,20)_ = 0.729, *p* = 0.403, *η*^2^ = 0.035) and the main effect for group (F_(1,20)_ = 0.031, *p* = 0.861, *η*^2^ = 0.002) were not statistically significant for force error in the PGT. However, there was a significant main effect for condition (F_(1,20)_ = 9.932, *p* = 0.005, *η*^2^ = 0.332), which indicated that force error was significantly lower in the Experimental condition compared to the Baseline condition (Figure 3A). Using the means and standard deviations from these analyses and the “repeated measures analysis” module on PASS 2020 (NCSS, LLC. Kaysville, UT, USA, www.ncss.com/software/pass), it was determined that 347 participants per group (336 additional participants per group) would be needed to achieve sufficient power to find a statistically significant interaction for the group × condition interaction for the PGT.

### 3.2. AMT

**Figure 4:**
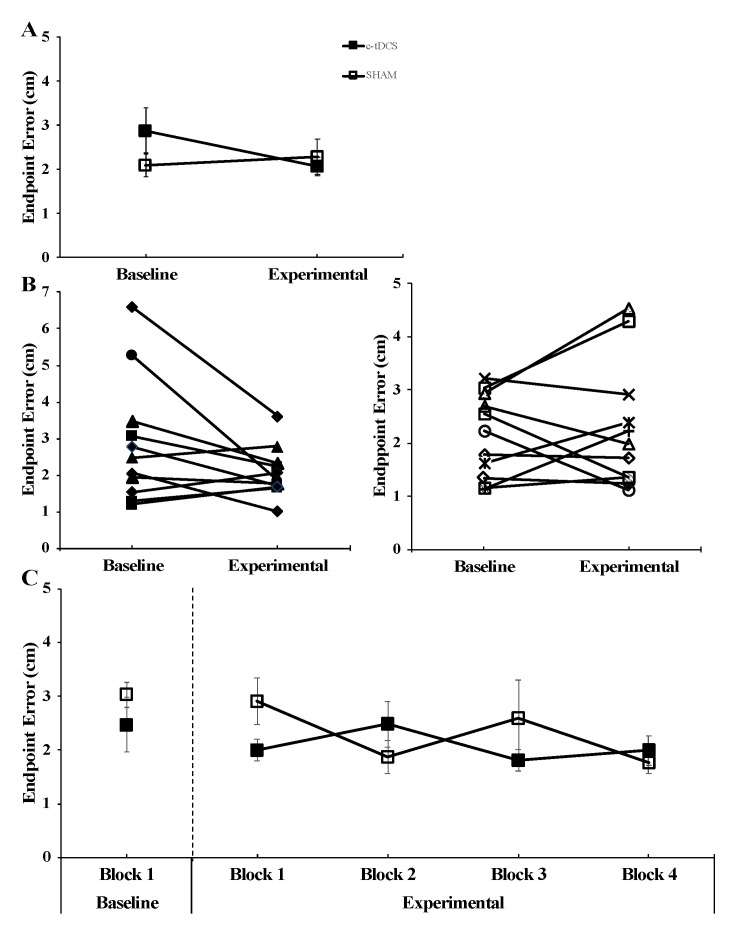
Endpoint error in the arm movement task (AMT) for the cerebellar transcranial direct current stimulation (c-tDCS) (closed squares) and SHAM groups (open squares) in the Baseline and Experimental conditions. (**A**) The endpoint error was not significantly different between c-tDCS and SHAM groups (*p* = 0.561) or for the Baseline and Experimental conditions (*p* = 0.164). (**B**) Individual endpoint error data corresponding to the data in Panel (A) for the 11 participants in the c-tDCS group and 11 participants in the SHAM group. (**C**) For illustrative purposes, the individual AMT trial blocks corresponding to the average data presented in Panel (A) are depicted. It can be seen that there is no trend for endpoint error to improve with trial block number to a greater extent in the c-tDCS group compared with the SHAM group due to the accumulating stimulation time.

For endpoint error in the AMT, the group × condition interaction was not statistically significant (F_(1,20)_ = 4.05, *p* = 0.058, *η*^2^ = 0.168). The main effect for group (F_(1,20)_ = 0.349, *p* = 0.561, *η*^2^ = 0.017) and main effect for condition (F_(1,20)_ = 2.092, *p* = 0.164, *η*^2^ = 0.095) were both non-statistically significant (Figure 4A). 

**Figure 5:**
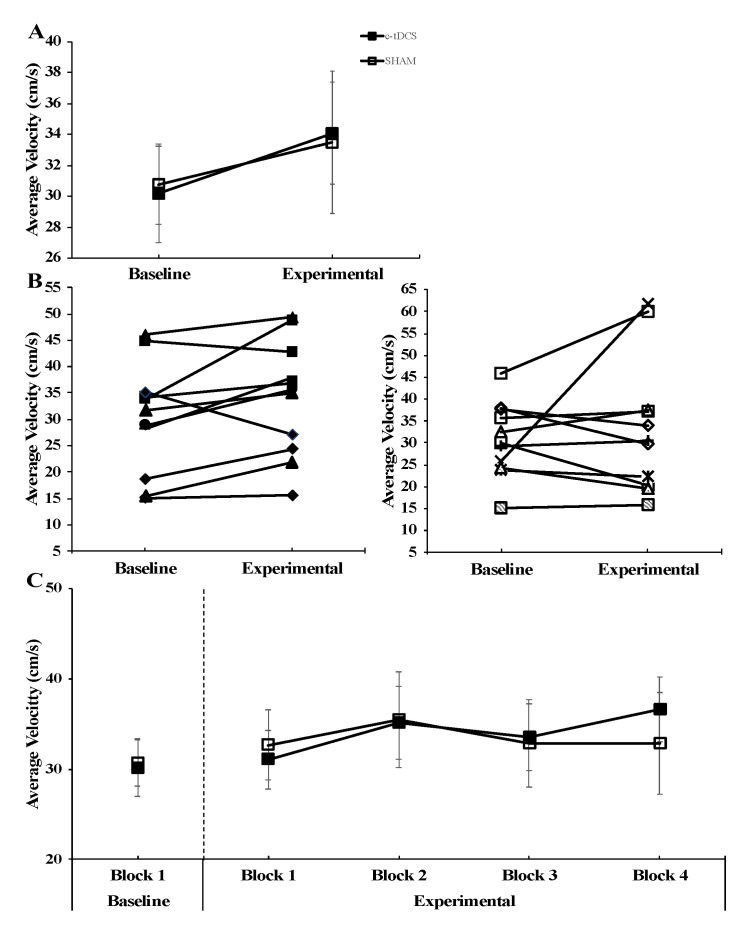
Average velocity for the arm movement task (AMT) for the cerebellar transcranial direct current stimulation (c-tDCS) (closed squares) and SHAM groups (open squares) in the Baseline and Experimental conditions. (**A**) The movement velocity was not significantly different between c-tDCS and SHAM groups (*p* = 0.994) or for the Baseline and Experimental conditions (*p* = 0.134). (**B**) Individual average velocity data corresponding to the data in Panel (A) for the 11 participants in the c-tDCS group and 11 participants in the SHAM group. (**C**) For illustrative purposes, the individual AMT trial blocks corresponding to the average data presented in Panel (A) are depicted. It can be seen that there is no trend for movement velocity to improve with trial block number to a greater extent in the c-tDCS group compared with the SHAM group due to the accumulating stimulation time.

Similarly, for average movement velocity in the AMT, the group × condition interaction was not statistically significant (F_(1,20)_ = 0.072, *p* = 0.791, *η*^2^ = 0.004). Finally, the main effect for group (F_(1,20)_ = 0.000, *p* = 0.994, *η*^2^ = 0.000) and main effect for condition (F_(1,20)_ = 2.443, *p* = 0.134, *η*^2^ = 0.109) were both non-statistically significant (Figure 5A).

## 4. Discussion

The purpose was to determine the effects of c-tDCS on motor performance in PD while participants were on medications. There were two main findings. First, c-tDCS did not significantly reduce force error in a complex, visuomotor precision grip task to a greater extent than SHAM stimulation. Second, c-tDCS did not significantly improve endpoint accuracy in a rapid, goal-directed arm movement task in PD. Collectively, these results indicate that a single session of c-tDCS does not elicit improvements in motor performance in hand and arm tasks in PD. 

### 4.1. Influence of Acute c-tDCS Application on Motor Performance in PD

c-tDCS administered simultaneously with the performance of motor tasks has been shown to elicit acute enhancements in motor performance in young and older adults. The capacity of tDCS to impact motor performance in older adults is particularly noteworthy for several reasons. First, accumulating evidence indicates that the cerebellum could be the predominant brain structure involved in the well-known movement impairments displayed by older adults [46]. Second, the majority of individuals with PD are older adults as the average age at initial diagnosis 60, which implies that at least some of the mechanisms of action of c-tDCS in old adults could generalize to PD. Third, the cerebellum is implicated in some of the motor control deficits exhibited by individuals with PD in arm movement tasks [42]. Specifically, cerebellar involvement is critical in multi-joint movement regulation related to factors such as the prediction and utilization, as well as the compensation for joint interaction torques [47,48], the modulation of antagonistic muscle interactions [26], and error detection in goal-directed tasks [27].

Based on these lines of reasoning and dysfunction of the cerebellum in PD, the present study examined the influence of c-tDCS on the performance of two motor tasks (PGT, AMT) in PD. The original hypothesis was that force error in the PGT and endpoint error in the AMT would be lower in the c-tDCS group compared with the SHAM group. Contrary to these expectations, the reduction in force error in the PGT from the Baseline session to the Experimental session was nearly identical between the two groups. For the AMT, the reduction in endpoint error was greater in the tDCS group, but this difference did not reach statistical significance due to the high degree of performance variability present in both groups of participants in this difficult arm movement task. Finally, endpoint error performance could not have been influenced by the velocity at which the AMT was performed as average movement velocity was similar between groups and between the Baseline and Experimental conditions.

Taken together, the results are not consistent with the majority of prior c-tDCS studies in young and old adults that also employed pinch grip and arm movement tasks [16,17,18,36]. For example, a recent study in our laboratory [18] found that motor skill acquisition was enhanced in a difficult overhand throwing task in young adults, when c-tDCS was given simultaneously with task practice. Most importantly, most of the effects of c-tDCS on motor skill were evident within the first block of 10 practice trials, but they continued at a decreasing rate until reaching the lowest point over the course of 20 further practice trials. The current results also differ from the augmentations in motor performance attained in the preponderance of M1-tDCS studies in PD [11,49,50]. Conversely, Steiner et al. found that c-tDCS did not increase the magnitude of motor learning attained by young adults in a whole-body dynamic balance task [51]. Furthermore, our results are highly compatible with a very similar c-tDCS study conducted in dystonia [52]. Specifically, Sadnicka et al. reported that one session of c-tDCS failed to improve clinical measures of handwriting skill and proficiency in dystonia [52]. These observations are applicable to the present study because dystonia is also predominantly a motor disorder of the basal ganglia, which, like PD, is associated with cerebellar dysfunction that negatively impacts movement control. Thus, c-tDCS may be ineffective at reducing motor impairments in these two particular movement disorders, at least when administered in a single, 25 min session. 

### 4.2. Possible Factors Responsible for the Lack Ability of c-tDCS to Improve Motor Performance in PD

The conflicting results of the current study relative to the vast majority of previous c-tDCS studies in young and older adults and M1-tDCS studies in PD suggest that care must be taken in assuming that c-tDCS always improves motor function or that findings obtained in healthy adults can always be extended to PD. Accordingly, there are several possible explanations that could explain the lack of ability of c-tDCS to enhance motor performance in the present task conditions. 

The most likely possibility is that administration of a single c-tDCS session is not adequate to substantially improve movement accuracy. Thus, repeated stimulation sessions over a time periods ranging from 3–10 days may be needed to induce noticeable effects in PD. Accordingly, this line of reasoning was identified as the most probable reason why c-tDCS did not improve clinical writing scales in dystonia [52]. Specifically, a strong case was made that it is highly doubtful that one c-tDCS session would be potent enough to supersede the long-term pathological changes associated with motor deficits in the disorder. Therefore, it is easily conceivable that this phenomenon was at least partially responsible for the current pattern of results in PD. These interpretations are supported by evidence from a series of M1-tDCS studies [33,34,53,54] as well as a prominent c-tDCS study [36], in motor learning effects accumulated over a timescale of 3–5 days. However, only one of these investigations found that the effects of tDCS on motor performance were not also significant on the first day of practice and stimulation. In addition, the overwhelming balance of tDCS studies in both healthy adults and in PD have only involved a single stimulation session and review articles [10,11,49,50] appear to show that tDCS yielded positive results in 75–80% of these acute studies. Accordingly, it is not obligatory that multiple daily stimulation sessions are required to observe enhancements in motor function due to c-tDCS. 

Another explanation for the current findings is that the several cerebellar-thalamic-cortical pathways that ultimately influence the corticospinal output cells in M1 display maladaptive changes in PD. Similarly, the recently identified bi-directional pathways [22] between cerebellum and basal ganglia almost certainly exhibit similar alterations. Thus, administration of c-tDCS in PD may not lead to the same modulation of behavioral outcomes in PD compared to those elicited in healthy adults. Accordingly, a number of transcranial magnetic stimulation (TMS) studies have demonstrated that several cerebellar pathways provide inputs onto interneuronal facilitatory and inhibitory circuits within M1 [55,56,57]. Therefore, the extensive dysfunction in all of the motor loops interconnecting the cerebellum, M1, and basal ganglia could potentially have contributed to the absence of improvements in motor performance found in the present study in PD. 

A final possibility is that c-tDCS parameters such as the electrode montage (electrode sizes, anode location, cathode location), the stimulation duration, and the current strength were not the ideal. Accordingly, some previous investigations have obtained significant results with stimulation parameters that differ in a few of these methodological aspects, at least in other populations and experimental contexts [58]. Although these assertions are conceivable, it seems very improbable as the exact same c-tDCS parameters were effective in enhancing motor skill acquisition in a previous study from our laboratory [18] in young adults and in a series of investigations by another research group in both young and older adults [16,17,59,60]. Nonetheless, a recent study by Workman and colleagues (2020) compared the effects of four c-tDCS electrode montage (unilateral, bilateral) and current intensity (2 mA, 4 mA) combinations on balance performance in PD [24]. The main finding was that only the bilateral montage that used a 4 mA current intensity improved balance scores in PD. Thus, it is possible that this montage and current intensity combination may be more effective for the improvement of motor performance in PD compared to the unilateral montage and 2 mA current intensity used in the present study. Future c-tDCS studies involving fine motor control tasks of the hand and arm in PD should investigate this possibility. Furthermore, it is unlikely that the two motor tasks used in current study are viable explanations for the lack of c-tDCS effects because a large number of studies have found that these tasks are highly amenable to c-tDCS and M1-tDCS and are characterized by high degrees of cerebellar involvement [23,33,34,36,53,61,62]. Nevertheless, all of the aforementioned possibilities will need to be investigated in additional studies that can simultaneously address the influence of c-tDCS applied over multiple days on the physiological mechanisms underlying behavioral outcomes in PD. 

## 5. Conclusions

In summary, the findings of the study indicated that a single session of c-tDCS does not improve motor performance in precision grip and arm movement tasks in PD. Thus, future investigations should determine the influence of repeated administration of c-tDCS over multiple days to further examine the utility of c-tDCS as a modality to enhance motor abilities in PD.
